# Environmentally Endemic *Pseudomonas aeruginosa* Strains with Mutations in *lasR* Are Associated with Increased Disease Severity in Corneal Ulcers

**DOI:** 10.1128/mSphere.00140-16

**Published:** 2016-09-07

**Authors:** John H. Hammond, Wesley P. Hebert, Amanda Naimie, Kathryn Ray, Rachel D. Van Gelder, Antonio DiGiandomenico, Prajna Lalitha, Muthiah Srinivasan, Nisha R. Acharya, Thomas Lietman, Deborah A. Hogan, Michael E. Zegans

**Affiliations:** a Department of Surgery (Ophthalmology), Geisel School of Medicine at Dartmouth, Hanover, New Hampshire, USA; b Department of Microbiology and Immunology, Geisel School of Medicine at Dartmouth, Hanover, New Hampshire, USA; c MedImmune, LLC, Gaithersburg, Maryland, USA; d FI Proctor Foundation and Department of Ophthalmology, University of California, San Francisco, San Francisco, California, USA; e Aravind Eye Hospital, Madurai, Tamil Nadu, India; Swiss Federal Institute of Technology Lausanne

**Keywords:** Anr, bacterial keratitis, corneal ulcer, CupA, eye infection, LasR, *Pseudomonas aeruginosa*, quorum sensing, SCUT

## Abstract

The Steroids for Corneal Ulcers Trial (SCUT) was a multicenter, international study of bacterial keratitis in which 101 *Pseudomonas aeruginosa* infections were treated. Twenty-two of 101 *P. aeruginosa* isolates collected had a colony morphology characteristic of a loss-of-function mutation in *lasR*, the gene encoding a quorum-sensing master regulator. Ulcers caused by these 22 strains were associated with larger areas of corneal opacification, worse vision, and a lower rate of vision recovery in response to treatment than ulcers caused by the other isolates. The *lasR* sequences from these isolates each contained one of three nonsynonymous substitutions, and these strains were deficient in production of LasR-regulated protease and rhamnolipids. Replacement of *lasR* with either of the two most common *lasR* alleles from the SCUT isolates was sufficient to decrease protease and rhamnolipid production in PA14. Loss of LasR function is associated with increased production of CupA fimbriae, and the LasR-defective isolates exhibited higher production of CupA fimbriae than LasR-intact isolates. Strains with the same *lasR* mutation were of the same multilocus sequence type, suggesting that LasR-deficient, environmental *P. aeruginosa* strains were endemic to the area, and infections caused by these strains were associated with worse patient outcomes in the SCUT study. (This study has been registered at ClinicalTrials.gov under registration no. NCT00324168.)

**IMPORTANCE** The LasR transcription factor is an important regulator of quorum sensing in *P. aeruginosa* and positively controls multiple virulence-associated pathways. The emergence of strains with *lasR* loss-of-function alleles in chronic disease is well described and is thought to represent a specific adaptation to the host environment. However, the prevalence and virulence of these strains in acute infections remain unclear. This report describes observations revealing that *lasR* mutants were common among isolates from a large, multicenter clinical study of keratitis and were associated with worse clinical outcomes than LasR-intact strains despite reduced production of LasR-regulated factors. Additionally, these *lasR* mutants were closely related strains or clones, as determined by molecular analysis. Because bacterial keratitis is community acquired, these data indicate infection by endemic, LasR-deficient strains in the environment. These results suggest that the conventional paradigm regarding the role for LasR-mediated regulation of virulence is more complex than previously appreciated.

## Abstract (precis)

The LasR transcription factor is an important regulator of quorum sensing in *P. aeruginosa* and positively controls multiple virulence-associated pathways. The emergence of strains with *lasR* loss-of-function alleles in chronic disease is well described and is thought to represent a specific adaptation to the host environment. However, the prevalence and virulence of these strains in acute infections remain unclear. This report describes observations revealing that *lasR* mutants were common among isolates from a large, multicenter clinical study of keratitis and were associated with worse clinical outcomes than LasR-intact strains despite reduced production of LasR-regulated factors. Additionally, these *lasR* mutants were closely related strains or clones, as determined by molecular analysis. Because bacterial keratitis is community acquired, these data indicate infection by endemic, LasR-deficient strains in the environment. These results suggest that the conventional paradigm regarding the role for LasR-mediated regulation of virulence is more complex than previously appreciated.

## INTRODUCTION

**FIG 1:**
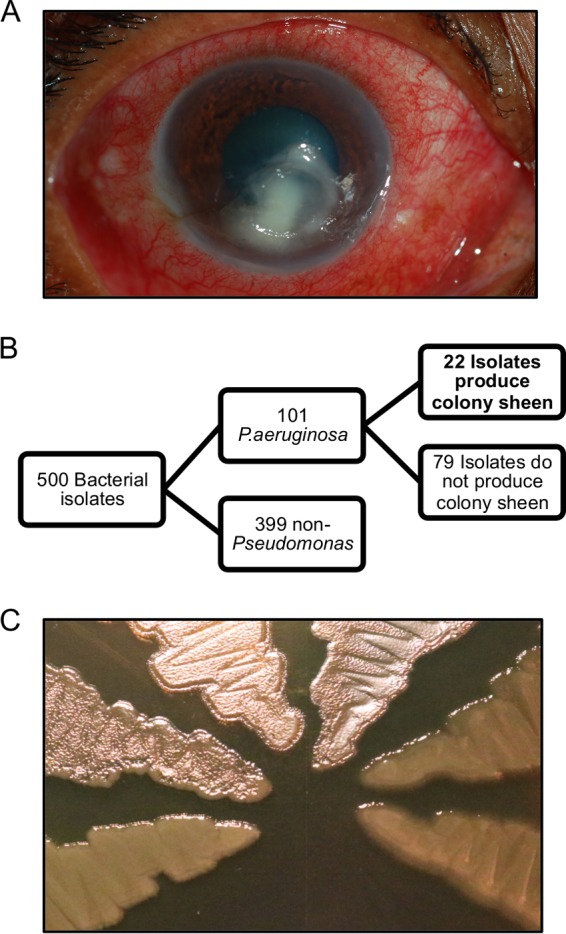
Isolates that exhibit the colony sheen phenotype represent a substantial percentage of *P. aeruginosa* clinical strains from the SCUT study. (A) Representative photo of *P. aeruginosa*-induced keratitis. Clinical strain 269F was isolated from the infection pictured. (B) Schematic of clinical isolate collection. (C) Colony sheen phenotype associated with *lasR* mutants as observed in SCUT clinical isolates. From left to right, the strains shown are PA14 wild type, PA14 Δ*lasR*, 262K, 283J, 265B, and 399D.

The Gram-negative environmental bacterium and opportunistic pathogen *Pseudomonas aeruginosa* is a major cause of corneal infection, also known as bacterial keratitis (Fig. 1A) (1, 2). *P. aeruginosa* can also cause acute infections at multiple other body sites and chronic infections in the lungs of individuals with cystic fibrosis (3).

*P. aeruginosa* coordinates the production of many cytotoxic factors in response to cell density via a signaling network termed quorum sensing (QS) (4). QS is a form of microbial cell-cell communication that operates through production of secreted, diffusible molecules that can bind and activate the transcription factors that regulate expression of QS-controlled pathways. In this way, bacterial cells in a population can simultaneously initiate the same genetic programs (5). The LasR transcription factor is a master regulator of QS in *P. aeruginosa*, and it is activated by 3-oxo-C12-homoserine lactone (3OC12HSL) generated by LasI (6). When in complex with 3OC12HSL, LasR positively regulates expression of secreted cytotoxic factors, including phenazines, proteases, and rhamnolipids (7).

*P. aeruginosa* clinical strains that are deficient in LasR-mediated signaling have long been detected in chronic infections, such as those that occur in cystic fibrosis (CF) (8), and the presence of *lasR* mutants in the airways of CF patients has been associated with worse disease progression (9). LasR-defective strains also emerge in more-acute airway infections (10) and in infections at other body sites (11). Loss-of-function mutations in *lasR* have been identified in *P. aeruginosa* environmental isolates, though this observation was limited to one study (12). Selection for loss or decrease in LasR function in infections is thought to be due to a combination of factors. In mixed cultures with LasR-intact strains, *lasR* mutants benefit from LasR-regulated, secreted public goods without suffering the energetic costs of producing these goods themselves, a phenomenon known as social cheating (13). *P. aeruginosa* strains without functional LasR also show growth advantages under certain nutritional conditions (14), resistance to cell lysis in high-density cultures (15), and increased resistance to oxidative stress and antibiotic treatment (14, 16). Decreased LasR signaling leads to increased expression of pathways regulated by the transcription factor Anr, a mediator of the cellular response to anoxia and hypoxia that plays roles in growth *in vivo* and in virulence factor regulation (17, 18).

The Steroids for Corneal Ulcers Trial (SCUT) study (ClinicalTrials registration no. NCT00324168) was conducted to evaluate topical corticosteroids as an adjunctive treatment to fluoroquinolone (moxifloxacin) in bacterial keratitis patients (19, 20). Among the 101 *P. aeruginosa* strains isolated from ulcers, 22 had phenotypes associated with severely decreased or absent LasR activity. Sequence analysis of *lasR* in these strains identified mutations predicted to negatively impact function, and the prediction was confirmed by replacing wild-type *lasR* with these *lasR* variant alleles in PA14. Three *lasR* variants were identified, and, surprisingly, strains with the same *lasR* sequence were also of the same multilocus sequencing type. These data suggested an environmental source for these LasR-defective strains, rather than rapid selection for LasR variants within the context of the acute infections. A comparison of levels of visual acuity and of ulcer size in patients found a striking relationship between LasR status and disease severity, with the LasR-deficient strains being associated with poorer outcomes.

## RESULTS

### *P. aeruginosa* clinical isolates exhibit a phenotype consistent with LasR loss of function.

Of the 500 patients with corneal infections enrolled in the SCUT study, 101 were infected with *P. aeruginosa* (Fig. 1B). Observation of the 101 isolates found that 22 produced an iridescent sheen on solid media such as is characteristic of strains with loss-of-function mutations in *lasR* (14) and that the remainder had a more classical *P. aeruginosa* colony phenotype (Fig. 1B and C). The iridescent material that causes the colony sheen morphology was previously identified as 2-heptyl-4-quinolone (HHQ), a signaling molecule itself and a precursor to the *Pseudomonas* quinolone system (PQS); HHQ accumulates in *lasR* mutants due to decreased expression of LasR-regulated *pqsH*, the gene encoding the enzyme that converts HHQ to PQS (14).

To test whether the clinical isolates with colony sheen were defective in LasR signaling, we sought to determine if they produced lower levels of the LasR-regulated 3OC12HSL signal. We assayed the ability of these clinical strains to induce β-galactosidase production by a reporter strain of *P. aeruginosa* (qsc102) that has *lacZ* under the control of LasR in a Δ*lasI* Δ*rhlI* background. In qsc102, *lacZ* is integrated into PA1897, a gene that is strongly controlled by the regulator QscR (21). QscR requires 3OC12HSL for activity, and the qsc102 reporter was shown previously to be activated specifically by exogenous 3OC12HSL (22). As expected, the *P. aeruginosa* strain PA14 wild type stimulated production of β-galactosidase in cross-streaks with qsc102, and its Δ*lasR* derivative did not (Table 1). Similarly to *P. aeruginosa* PA14 Δ*lasR*, all of the clinical isolates that had a colony sheen phenotype also failed to induce β-galactosidase (Table 1). Ten randomly selected SCUT *P. aeruginosa* isolates that do not produce colony sheen induced β-galactosidase activity similarly to *P. aeruginosa* strain PA14. These findings suggest that strains deficient in LasR signaling represent a substantial portion (21.8%) of the *P. aeruginosa* bacterial keratitis isolates in the SCUT study.

**TABLE 1:** Characterization of QS-regulated phenotypes, LasR coding sequence, and MLST type in clinical isolates and laboratory strains^a^

*P. aeruginosa* strain	Colony sheen	Activation of 3OC12-HSL reporter	BHI-milk agar hydrolysis (mm) (±SD)	Drop collapse activity^b^	LasR substitution^c^	MLST sequence type	Isolation site^d^
PA14 WT	−	+	8.17 ± 0.29	1:16	None		
PA14 Δ*lasR*	+	−	3.67 ± 0.29	1:8			
283J	+	−	1.67 ± 0.29	U	P117L	155	2
295K	+	−	1.83 ± 0.29	U	P117L	155	2
360J	+	−	1.67 ± 0.29	U	P117L	155	1
362M	+	−	1.83 ± 0.29	U	P117L	155	1
364B	+	−	2.67 ± 0.29	U	P117L	155	1
367E	+	−	2.00 ± 0.00	U	P117L	155	1
369G	+	−	2.50 ± 0.50	U	P117L	155	1
262K	+	−	1.33 ±. 0.29	1:8	I215S	244	2
269F	+	−	1.00 ± 0.00	1:8	I215S	244	2
271H	+	−	1.33 ± 0.29	1:8	I215S	244	2
282H	+	−	1.33 ± 0.29	1:8	I215S	244	2
286A	+	−	1.00 ± 0.00	1:8	I215S	244	2
288C	+	−	1.33 ± 0.29	1:8	I215S	244	2
376C	+	−	1.17 ± 0.29	1:8	I215S	244	1
378E	+	−	1.17 ± 0.29	1:8	I215S	244	1
385A	+	−	1.17 ± 0.29	1:8	I215S	244	1
388D	+	−	1.50 ± 0.50	1:8	I215S	244	1
403H	+	−	1.17 ± 0.29	1:8	I215S	244	1
404J	+	−	0.83 ± 0.29	1:8	I215S	244	1
406M	+	−	1.00 ± 0.00	1:8	I215S	244	1
417M	+	−	1.00 ± 0.00	1:8	I215S	244	1
419B	+	−	3.33 ± 0.29	1:8	V221L	316	1
399D	−	+	6.33 ± 0.29	1:16	None	381	1
265B	−	+	7.33 ± 0.29	1:16	None	381	2
550A	−	+	8.00 ± 0.50	1:16	None	244	3
654F	−	+	6.67 ± 0.29	1:16	None	379	3
904C	−	+	7.33 ± 0.29	1:16	None	17	4
352A	−	+				381	1
432D	−	+				274	1
123C	−	+				1197	2
321C	−	+				769	2
901M	−	+				253	4
PA14 LasR P117L	+	−	3.67 ± 0.29	1:8	P117L		
PA14 LasR I215S	+	−	3.33 ± 0.29	1:8	I215S		
WT revert	−	+	7.83 ± 0.29	1:16	None		

a BHI, brain heart infusion; revert, revertant; WT, wild type.

b Data represent the dilutions of filtered supernatant at which drop collapse was not observed. U, undiluted.

c LasR substitution, amino acid change relative to the PA14 protein sequence.

d Site key: 1, Coimbatore, India; 2, Madurai, India; 3, Pondicherry, India; 4, Lebanon, NH, USA.

### Clinical isolates that produce colony sheen produce lower levels of LasR-secreted products.

When bound to 3OC12HSL, LasR positively regulates expression of *lasB*, a gene encoding a metalloprotease with destructive activity against host tissues (23, 24). To evaluate protease production by the SCUT isolates, we measured the zone of clearance produced on agar plates with brain heart infusion and skim milk (25). The sheen-producing isolates had a statistically significantly smaller zone of proteolysis than strain PA14 or the clinical isolates that lacked colony sheen (*P* < 0.001 as determined by unpaired *t* test) (see Table 1 for data).

*P. aeruginosa* LasR regulates production of surfactants known as rhamnolipids that play roles in surface motility (26), biofilm formation (27), and immunomodulation of the host immune response (28). We evaluated rhamnolipid production using a drop collapse assay (29) and found that production of rhamnolipids was significantly reduced in sheen-producing isolates compared with PA14 and non-sheen-producing SCUT strains (*P* < 0.0001 as determined by Mann-Whitney test) (Table 1). Taken together, these data confirm that the sheen-producing isolates from the SCUT study were also deficient in production of protease and rhamnolipids, consistent with LasR loss of function.

### Sequence analysis of the *lasR* allele from sheen-producing isolates.

To determine if the observed defects in QS-regulated pathways are due to differences in *lasR* primary sequences, the *lasR* coding sequence and 225 bp upstream of *lasR* translational start were sequenced in all 22 sheen-producing isolates and in 5 nonsheen isolates. Interestingly, we found that all of the strains that displayed a colony sheen phenotype had one of three *lasR* alleles (Table 1). In 14 of the isolates, *lasR* contained a T644G transversion encoding a LasR I215S variant (located in the DNA binding domain of the protein). In 7 of the isolates, *lasR* contained a C350T transition encoding LasR P117L, located in the dimerization domain. One of the isolates contained a *lasR* G661T transversion encoding a LasR V221L variant. The 5 randomly selected isolates that did not produce colony sheen had *lasR* sequences identical to *lasR* sequences from strains PAO1 and PA14.

### The LasR P117L and I215S alleles are sufficient to disrupt production of LasR-regulated products in PA14.

To confirm that the most commonly observed LasR variants, P117L and I215S, were loss-of-function variants, we replaced the gene encoding native LasR with either the P117L or 1215S LasR allele in PA14 and evaluated these strains in the assays for colony sheen, protease production, and rhamnolipid production described above (Table 1). We observed that the P117L or I215S LasR variant was sufficient for production of colony sheen. We also observed a reduction of clearance in the proteolysis assay compared to the results seen with the wild-type parental strain or a wild-type revertant from sucrose counterselection of the merodiploid. No differences between the PA14 LasR P117L, PA14 LasR I215S, and PA14 Δ*lasR* isolates were observed in the protease assay or the drop collapse assay for rhamnolipid production. These data indicate that the P117L and I215S substitutions in LasR decrease or abolish LasR activity and that the presence of these *lasR* alleles in the SCUT clinical isolates was sufficient to explain the colony sheen phenotype and the decreased production of protease and rhamnolipids in these isolates.

### *lasR* mutants produce large amounts of CupA fimbriae relative to LasR-intact strains.

**FIG 2:**
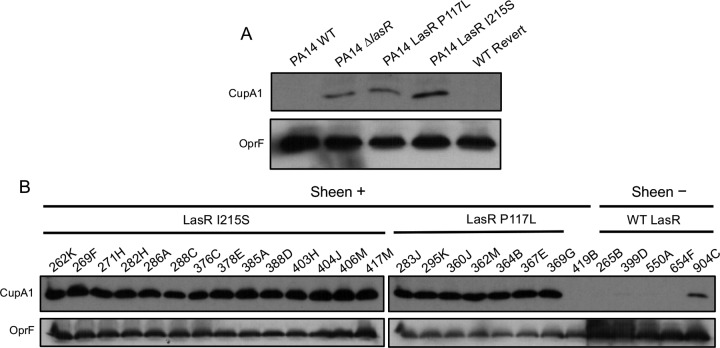
Clinical isolates and laboratory strains with LasR loss-of-function mutations produce increased amounts of CupA fimbriae. Levels of production of CupA1 and OprF, a reference protein, were determined by Western blotting of whole-cell lysates from 14-h colony biofilms grown on T-broth agar in 0.2% O_2_. (A) PA14 wild-type (WT) and isogenic derivatives. Revert, revertant. (B) SCUT clinical isolates organized by presence or absence of colony sheen and by *lasR* variant type.

We recently reported that both genetically constructed and naturally occurring *lasR* mutants had increased production of CupA fimbriae compared with paired strains with functional LasR (17). The CupA fimbriae have been implicated in cell-cell and cell-surface interactions in *P. aeruginosa* (30, 31), and type I fimbriae were shown to be a critical adhesin for attachment to cultured human corneal epithelial cells in *Serratia marcescens* (32). In *P. aeruginosa*, CupA is necessary for persistence in both chronic and acute models of infection (33, 34), although the specific role for CupA in disease is not well understood. To further assess the activities of the LasR variants identified in the SCUT isolates, we measured CupA1 protein levels in colony biofilms via immunoblotting performed with a polyclonal anti-CupA1 antibody (35). We observed that introduction of LasR P117L and I215S into PA14 was sufficient to induce CupA1 production (Fig. 2A). We also found that all LasR-deficient isolates showed high levels of CupA1 production compared to strains with native LasR, with the exception of 419B (LasR V221L) (Fig. 2B). Thus, both PA14 and the SCUT isolates with *lasR* alleles encoding the LasR P117L and I215S variants have the described characteristics that accompany the absence of LasR function in *P. aeruginosa*, including increased production of CupA fimbriae and decreased production of proteases, rhamnolipids, and 3OC12HSL.

### Multilocus sequence typing of LasR-deficient *P. aeruginosa* isolates reveals the presence of three sequence types.

**FIG 3:**
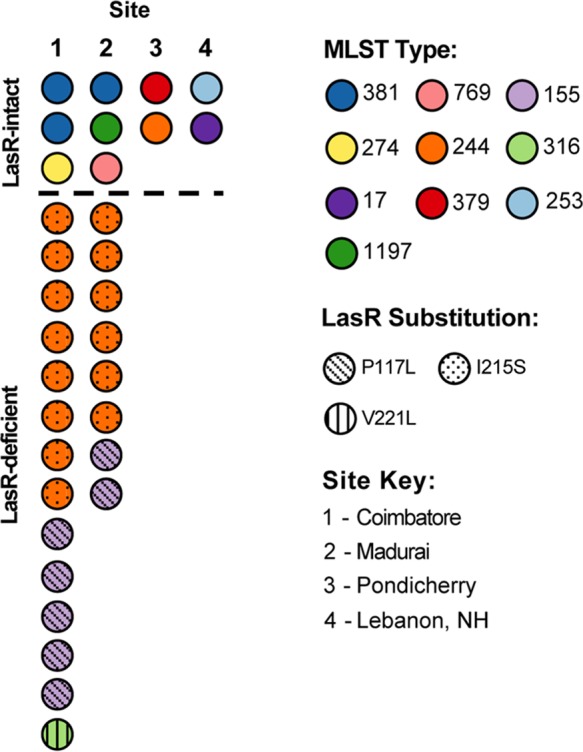
MLST diversity is greater among LasR-intact strains than among LasR-deficient strains in the SCUT study, and LasR-intact and LasR-deficient strains from the same study sites have different MLST assignments.

In light of numerous reports of LasR-deficient isolates arising within infections and of characterization of many LasR loss-of-function variants, we were struck by the presence of identical LasR variants (P117L and I215S) caused by identical DNA mutations (C350T and T644G, respectively) in the SCUT study. This was especially surprising because strains with either variant were isolated from two different study sites (Table 1) over the course of 2 years. Thus, we sought to determine the relatedness of these isolates using multilocus sequence typing (MLST), in which regions of *acsA*, *aroE*, *guaE*, *mutL*, *nuoD*, *ppsA*, and *trpE* were sequenced. We found that all isolates with LasR I215S belonged to *P. aeruginosa* sequence type 244 (ST-244) and that all isolates with LasR P117L belonged to ST-155 (Table 1). The single isolate with a V221L LasR variant belonged to a third sequence type, ST-316. Only three of the seven loci sequenced in the MLST analysis were identical in the ST-155 and ST-244 strains (*aroE*, locus 5; *mutL*, locus 3; *trpE*, locus 7), and ST-316 was identical to only one other ST at the *mutL* locus (*mutL*, locus 3), indicating that the three sequence types identified are not closely related. MLST was also used to assign sequence types to 10 LasR-intact strains that were positive for 3OC12HSL production and negative for colony sheen, comprising the 5 strains analyzed for their QS-related phenotypes as described above as well as an additional 5 strains from study sites 1, 2, and 4 (Coimbatore, India, Madurai, India, and Lebanon, NH, USA, respectively) (Table 1). The LasR-intact strains had a higher diversity of MLST types than the LasR-deficient strains (8 of 10 strains had unique MLST assignments in the LasR-intact group, whereas 21 of 22 strains from the LasR-deficient group belonged to sequence type 155 or 244). These data are consistent with the model proposing that there are specific lineages in the environment that have lost normal LasR function. Note that LasR-intact isolate 550A also belongs to ST-244. This strain was isolated from study site 3 (Pondicherry, India), whereas all of the LasR-deficient isolates were collected at study sites 1 and 2. This may suggest that the presence of closely related strains or clones with *lasR* loss-of-function mutations in the environment was specific to a certain geographical region. The results of the MLST studies are summarized in Fig. 3.

To use a more sensitive measure to analyze the relatedness among isolates with common LasR variants and MLST types, the isolate DNAs were analyzed by enterobacterial repetitive intergenic consensus-PCR (ERIC-PCR) (see Fig. S1 in the supplemental material). Among the isolates with LasR P117L, there were clear dissimilarities, with 283J and 360J being similar to each other and distinct from the rest of the P117L isolates. The ERIC-PCR profiles among the I215S isolates were more uniform. Consistent with the MLST data which showed that P117L- and I215S-bearing strains were of different sequence types, there were striking differences between the P117L and I215S groups and between the SCUT isolates and laboratory strains PAO1 and PA14 (see Fig. S1). The presence of SCUT isolates from different subjects at different centers with *lasR* alleles, multilocus sequence types, and ERIC-PCR profiles in common suggests that there are environmental sources of closely related, LasR-defective strains and that the patients that presented with keratitis in the SCUT study were infected with *P. aeruginosa* strains containing *lasR* loss-of-function alleles that were endemic in the environment.

10.1128/mSphere.00140-16.1 Figure S1  ERIC-PCR of genomic DNA. (A) Clinical isolates possessing the LasR P117L allele. (B) Clinical isolates possessing the I215S allele. L, 1 kb+ ladder from Invitrogen. *P. aeruginosa* strains PAO1 and PA14 were included on each gel for comparison. Download Figure S1, PDF file, 0.4 MB. Copyright © 2016 Hammond et al. 2016 Hammond et al. This content is distributed under the terms of the Creative Commons Attribution 4.0 International license.

### SCUT isolates with *lasR* loss of function are associated with worse patient outcomes.

**FIG 4:**
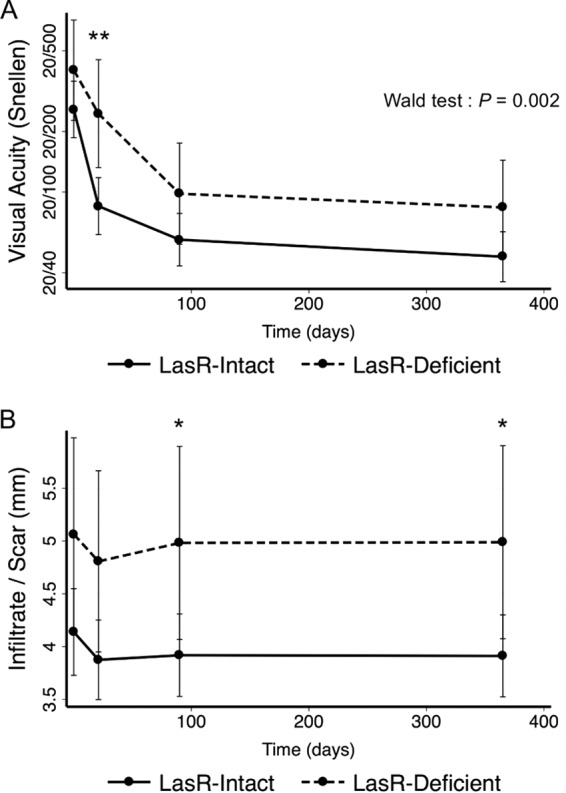
LasR mutants were associated with worse clinical outcomes in the SCUT study. Each point represents a study visit. Data points are estimated marginal means based on the GEE model, and error bars represent 95% confidence intervals. Significance was measured using Stata margins command (58). *, *P* < 0.05; **, *P* < 0.01. (A) Visual acuity. Data were converted from logMAR values to Snellen values for ease of interpretation. For reference, 20/20 Snellen = 0.0 logMAR, 20/40 Snellen = 0.3 logMAR, 20/200 Snellen = 1.0 logMAR, Snellen 20/20 = “normal” vision, Snellen 20/40 = legal driving requirement (United States), Snellen 20/200 = legal blindness. The Wald test was used to measure the rate of improvement. (B) Infiltrate/scar size. Units (in millimeters) refer to the geometric mean of the longest dimension of the scar/infiltrate and the longest dimension that is perpendicular to the first measurement (in millimeters) (36).

Because LasR is known to regulate virulence factor production in *P. aeruginosa*, we sought to determine whether *lasR* mutants were associated with differences in clinical outcomes. Surprisingly, patients infected with LasR-deficient *P. aeruginosa* isolates had a statistically significant lower rate of improvement in visual acuity than those infected with LasR-intact strains as determined by a Wald test (*P* = 0.002) (Fig. 4A). Retrospective analysis found that patients infected with *lasR* mutants (*n =* 22) had worse vision at all four study visits than those infected with non-sheen-producing, LasR-intact isolates (*n* = 79; Fig. 4A). Visual acuity was worse in the patients with *lasR* mutants at enrollment (0.19 logMAR; 95% confidence interval [CI], −0.091 to 0.48; *P* = 0.18) and at 3 weeks (0.46 logMAR; 95% CI, 0.16 to 0.76; *P* = 0.003), 3 months (0.23 logMAR; 95% CI, −0.05 to 0.51; *P* = 0.12), and 12 months (0.24 logMAR; 95% CI, −0.02 to 0.51; *P* = 0.07).

At enrollment, patients with LasR-deficient isolates had an infiltrate or scar that was 0.93 mm larger in size than those seen with patients with LasR-intact isolates (95% CI, −0.07 to 1.93; *P* = 0.07) (Fig. 4B). The infiltrate/scar size remained worse (i.e., larger) for those patients at future visits, at 3 weeks (0.93 mm; 95% CI, −0.004 to 1.87; *P* = 0.051), 3 months (1.06 mm; 95% CI, 0.07 to 2.06; *P* = 0.04), and 12 months (1.08 mm; 95% CI, 0.08 to 2.07; *P* = 0.03) (“mm” refers to the geometric mean of the longest dimension of the scar/infiltrate and the longest dimension that is perpendicular to the first measurement, in millimeters) (36). There was no evidence of a difference in the rates of infiltrate/scar size reduction over time between the two groups (*P* = 0.71 as determined by a Wald test).

The combination of a significantly lower rate of vision improvement with significantly worse vision at 3 weeks and significantly larger scar/infiltrate sizes at 3 months and 12 months in the LasR-deficient group supports the conclusion that *lasR* mutants were associated with increased disease severity in the SCUT study.

## DISCUSSION

**FIG 5:**
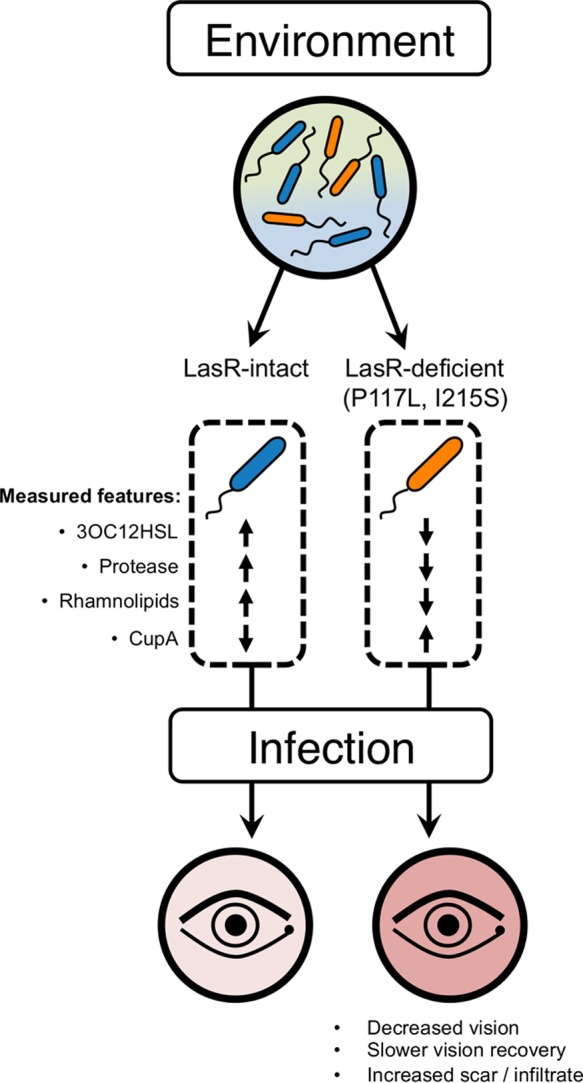
*P. aeruginosa* clones or closely related strains that are deficient in LasR signaling persist in the environment and cause keratitis. *P. aeruginosa* strains are present in the environment both with and without functional LasR. Strains with disrupted LasR signal from this study (LasR P117L and I215S) show reduced production of protease and rhamnolipids and increased production of CupA fimbriae and are associated with worse patient outcomes.

In the present study, we discovered that 22 of 101 *P. aeruginosa* clinical isolates from the SCUT study had mutations in the QS master regulator *lasR.* These *lasR* mutants exhibited the predicted deficiencies in production of secreted factors that are regulated by the QS signaling cascade, and we showed that the LasR loss-of-function variants identified in the SCUT isolates were associated with increased production of CupA fimbriae. Patients infected with the *lasR* mutants from this study experienced worse clinical outcomes than patients infected with *lasR*-intact strains, and we determined that isolates with *lasR* mutant alleles in common are either closely related strains or clones (Fig. 5).

Our finding that the LasR-deficient isolates caused worse disease is intriguing in light of the fact that LasR-regulated factors are thought to contribute to *P. aeruginosa* pathogenicity (37). Most commonly, the selection for LasR-deficient strains has been reported within the context of chronic infections such as cystic fibrosis (8, 9). In this setting, loss of LasR is thought to be part of a selective adaptation to the unique environment of prolonged pulmonary infection. However, there were previous studies in both ocular and nonocular settings that suggested that *lasR* mutants are capable of establishing acute infections. In a murine corneal-scratch model, no significant difference was observed between wild-type *P. aeruginosa* and a *lasR* mutant in infection rate or in scored severity of infection (38). The report also identified two contact lens isolates which were *lasI* and *lasR* negative, consistent with our finding that *lasR* mutations can arise outside the setting of chronic infections (38). A previous study using the same model found that wild-type *P. aeruginosa* and a *lasR* mutant were recovered in similar numbers from the cornea following infection (39) and that, additionally, the LasR-defective strain had a 10-fold-lower 50% infectious dose (ID_50_) than the wild type (39). In another study using the murine pneumonia model of acute infection, a Δ*lasRI* Δ*rhlRI* mutant was not attenuated (40). Our data support the hypothesis that LasR-mediated signaling is nonessential for *P. aeruginosa* pathogenicity in the context of corneal infection and that *lasR* mutants can cause disease despite decreased production of LasR-regulated, pathogenesis-associated products.

Production of CupA fimbriae in *P. aeruginosa* is regulated by multiple factors, including the oxygen-sensitive transcription factor Anr, which is a strong positive regulator of CupA (17, 41), as well as of multiple pathways that allow *P. aeruginosa* to generate energy under low-oxygen conditions (42, 43, 59). Anr is also necessary for expression of conventional virulence-associated pathways, including transcripts related to iron acquisition and storage, quinoline production, and type VI secretion (17), and we previously demonstrated a role for Anr in persistence in a model of airway infection (18). We recently reported that constructed and naturally occurring *lasR* mutants showed higher expression of transcripts regulated by Anr than paired strains with wild-type LasR and that Anr is required for CupA production in both LasR-intact and LasR-deficient strains (17). Increased expression of Anr-regulated proteins, including CupA, may contribute to selection for the LasR-deficient isolates in the SCUT study and to the ability of these isolates to cause infections.

CupA production is also strongly influenced by the histone-like nucleoid structuring (HNS) proteins MvaT and MvaU (30), which repress multiple genes associated with virulence (44), and by intracellular levels of cyclic diguanylate (35). Future studies will determine the relative contributions of Anr, HNS, and cyclic diguanylate to CupA production and virulence in the LasR-deficient SCUT isolates.

The prevalence of *lasR* mutants observed in the SCUT study suggests that these mutants may be more common in corneal disease than previously thought. This is consistent with a previous study comparing transcriptional profiles of *P. aeruginosa* clinical isolates using transcriptome sequencing (RNA-Seq), which found that 48 of 151 isolates (32%) contained inactivating mutations in LasR (45). A separate analysis of 66 clinical isolates and environmental strains found that 20% had nonsynonymous substitutions or large insertions in LasR (12). The same study determined that one environmental strain and one clinical isolate contained identical amino acid substitutions in LasR (R66K, N136S, A137N, G172N). In an earlier publication, these two isolates were shown to be closely related but not identical strains (46). These findings are very similar to what we found in the LasR P117L or P1215S isolates from the SCUT study.

Our observation that the P117L or I215S variants were caused by identical DNA mutations in all strains that shared each given variant strongly suggests that these mutations did not arise as a result of selective pressure *in vivo.* Epidemic clones of *P. aeruginosa* have been identified in instances where patients were linked by a common clinical source (47–49), but there is substantial literature describing the isolation of infectious clones that are unconstrained geographically or temporally, as we understand to be the case for the 22 SCUT isolates described here (50–53). Because the SCUT sample collection protocol called for only one isolate per patient, we do not know whether the *lasR* mutants reported here were present in mixed infections with LasR-intact strains. Recent work has demonstrated that hydrogen cyanide produced by LasR-intact *P. aeruginosa* suppresses expansion of social cheats that have lost functional LasR (54). Interestingly, this system of control would be nonapplicable in an infection that was homogenously LasR deficient. Taken together, our data are consistent with the hypothesis that endemic clones deficient in LasR signaling represent persistent populations in the environment that are capable of causing infections.

This study established that *lasR* mutants represent a substantial percentage of all *P. aeruginosa* clinical isolates collected during the SCUT study, that highly related strains with identical *lasR* loss-of-function mutations may persist in the environment over time, and that these isolates were associated with more-severe disease compared with a set of LasR-intact strains from the same study. Our data also indicate that the specific *lasR* mutations observed in these strains are sufficient to induce production of CupA fimbriae, which are virulence associated in other models of infection and whose presence may be indicative of increased activity of the transcription factor Anr. Future work will elaborate on the prevalence of *lasR* mutants in keratitis patient samples and on the relationships between LasR and virulence in these strains.

## MATERIALS AND METHODS

### Study description.

Between 1 September 2006 and 22 February 2010, 500 patients were enrolled in the study. More than 90% of subjects were enrolled at the multicenter Aravind Eye Care System in Tamil Nadu, India, with the remaining subjects enrolled at the University of California, San Francisco, and the Dartmouth-Hitchcock Medical Center. The isolates described here were collected between 2007 and 2009. Assessments of corneal infiltrate/scar size and visual acuity were performed on patients at enrollment and at 3-week, 3-month, and 12-month visits. Visual acuity and scar/infiltrate size were measured as described in reference 36. Snellen and logMAR charts are different charts for determining visual acuity. Data for this study were collected using the logMAR chart, and all data displayed in Snellen values were converted from logMAR values. Baseline measurements of infiltrate/scar size in each infection were taken prior to identification of infecting bacteria and prior to our characterizations of *P. aeruginosa* clinical isolates. One isolate was collected per infection, and those working on the laboratory assessment of isolates were blind to the clinical data associated with the subjects from which these strains were isolated. The Institutional Review Board of the Aravind Eye Care System, the Dartmouth-Hitchcock Medical Center Committee for the Protection of Human Subjects, and the Committee on Human Research of the University of California, San Francisco, gave institutional review board approval for the SCUT study. Written informed consent was obtained from all participants. The trial adhered to the Health Insurance Portability and Accountability Act and the tenets of the Declaration of Helsinki and was registered at clinicaltrials.gov (ClinicalTrials registration no. NCT00324168).

### Bacterial strains and growth conditions.

All strains used in this study are listed in Table S1 in the supplemental material. Colony sheen was observed after 36 h of incubation at 37°C on lysogeny broth (LB) agar plates. The LasR-intact isolates were randomly selected by sequentially numbering all isolates that did not produce colony sheen with the numbers 1 to 79 and using the RANDBETWEEN function in Microsoft Excel to randomly generate numbers between 1 and 79. The isolates corresponding to the numbers returned were the ones used. Bacteria were routinely grown with shaking at 37°C in LB medium. For colony biofilms, 1 ml of overnight culture was washed in T-broth (10 g tryptone and 5 g NaCl per liter) and diluted to an optical density at 600 nm (OD_600_) of 1.0. Six 5-µl inocula were spotted onto T-broth agar plates and incubated at 37°C inside a hypoxic cabinet with an O_2_ controller and a CO_2_ controller (Coy Laboratory Products, Grass Lake, MI) at 0.2% O_2_ and 5% CO_2_. The resultant colonies were harvested by suspension in phosphate-buffered saline (PBS) followed by scraping with a glass rod.

10.1128/mSphere.00140-16.2 Table S1  Strains, plasmids, and primers used in the study. Download Table S1, DOCX file, 0.1 MB. Copyright © 2016 Hammond et al. 2016 Hammond et al. This content is distributed under the terms of the Creative Commons Attribution 4.0 International license.

### Genomic DNA isolation and PCR amplification, purification, and sequencing.

Genomic DNA was isolated by using the Qiagen (Gentra Puregene) protocol and amplified using Phusion High-Fidelity DNA polymerase (New England Biolabs) with primers *lasR*_seq_For and *lasR*_seq_Rev*.* Amplicons were sequenced at the Dartmouth College Molecular Biology Shared Resource Sequencing core using these same primers. ERIC-PCR was performed using primers ERIC1R and ERIC2 as previously described in reference 55.

### Construction of plasmids and LasR substitution mutants.

*P. aeruginosa* strains PA14 LasR P117L and PA14 LasR I215S, bearing the two *lasR* alleles common among the LasR-defective strains, were generated via allelic exchange using a *Saccharomyces cerevisiae* recombination technique and the pMQ30 allelic replacement vector as reported previously (56). Amplicons containing LasR P117L or LasR I215S were generated by amplifying the *lasR* coding sequence from strain 283J or strain 262K, respectively, using primers *lasR*_KON_pMQ30_3 and *lasR*_KON_pMQ30_4. Fragments comprising 1,000 bp upstream and downstream of *lasR* were amplified from wild-type PA14 using primers *lasR*_KON_pMQ30_1 and *lasR*_KON_pMQ30_2 and primers *lasR*_KON_pMQ30_5 and *lasR*_KON_pMQ30_6, respectively.

### Acyl-homoserine lactone production assay.

Strains were tested for HSL production on LB plates containing 150 µg/ml X-Gal (5-bromo-4-chloro-3-indolyl-β-d-galactopyranoside). Plates were streaked with 3OC12HSL reporter strain qsc102 (22) and incubated for 8 h at 37°C, at which point the strains to be tested were struck through qsc102 and grown overnight at 37°C. Plates were transferred to room temperature and monitored until the PA14 Δ*lasR* mutant showed faint blue coloring due to X-Gal cleavage. At this point (roughly 48 h), the assay was considered complete and any strain with increased β-galactosidase production compared with the Δ*lasR* mutant results was recorded as positive for 3OC12HSL production.

### Protease activity assay.

Protease production by *P. aeruginosa* colonies was measured using an adaptation of a previously published method (25). Plates were prepared with 1.5% skim milk, 1.5% agar, and 37 g/liter of brain heart infusion. Overnight cultures (3 µl) were spotted on plates and incubated at 37°C for 36 h. The distance from the edge of the colony to the edge of the zone of clearance was measured to evaluate protease production. Values reported represent averages of the results from 3 biological replicates.

### Drop-collapse assay.

Surfactant production was measured using a modified drop-collapse assay (29). A pipette was used to spot the surface of overturned sterile Corning 96-well lids (Costar 3595; Corning, NY) with 3-µl aliquots of 10W-40 Pennzoil motor oil, and the spots were dried overnight at room temperature. Isolates to be tested were grown in PPGAS media (0.02 M NH_4_Cl, 0.02 M KCl, 0.12 M Tris-HCl, 0.0016 M MgSO_4_, 1% Proteose peptone [Difco], 0.5% glucose) at 30°C for 16 h, at which point cells were pelleted by centrifugation and the supernatant was removed and filtered using 0.22-µm-pore-size syringe filters (Millipore). Each filtered supernatant to be tested was serially diluted by factors of 2 in water, and 10-µl aliquots of each dilution were spotted on the dried Pennzoil spots using a multichannel pipette. The dilution reported is the dilution at which, due to surface tension, drop collapse did not occur.

### Western blot analysis of CupA1.

Samples were prepared from colony biofilms grown as described above. Pellets were suspended in loading buffer and boiled to generate a whole-cell lysate. Protein concentrations were quantified using a bicinchoninic acid (BCA) protein assay reagent (Pierce Biotechnology, Inc.), and 20 µg total protein was electrophoresed on a 10% SDS-PAGE gel. Separated proteins were transferred to a polyvinylidene difluoride (PVDF) membrane using a transblot Turbo Transfer system from BioRad, and the membrane was probed for CupA1 or OprF as previously described (35, 57).

### Multilocus sequence typing of clinical isolates.

Multilocus sequencing of all *lasR* mutants was performed by ID Genomics (Seattle, WA). The DNA sequences of *acsA*, *aroE*, *guaE*, *mutL*, *nuoD*, *ppsA*, and *trpE* were determined.

### Statistical analyses.

We compared clinical outcomes (visual acuity and infiltrate/scar size) by LasR status (patients infected by LasR-deficient strains versus patients infected by LasR-intact strains) for all four study visits using generalized estimating equation (GEE) models with an exchangeable correlation structure. The GEE models with repeated measures of the clinical outcomes were clustered by patient and included terms for LasR status, study visit, and interaction between LasR status and study visit. The differences in means between the LasR groups at each time point were predicted using postestimation computations with the Stata 13.1 margins command (58). A Wald test, which compared the rates of visual acuity or infiltrate/scar improvement over time between the groups, was used to determine the significance of the interaction term. A *P* value of less than 0.05 indicated that the clinical improvement in visual acuity or reduction in scar size increased faster in one group than in another group at a statistically significant rate. Visual acuity quantified with logMAR values was used for statistical analysis.
