# Supplementary figures and images for: Environmentally Endemic Pseudomonas aeruginosa Strains with Mutations in lasR Are Associated with Increased Disease Severity in Corneal Ulcers
Source: mSphere. 2016 Sep 7;1(5):e00140-16. doi: 10.1128/mSphere.00140-16 (PMC5014915; doi:10.1128/mSphere.00140-16)

A

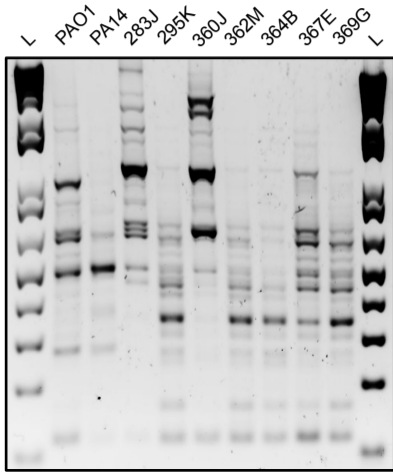

B

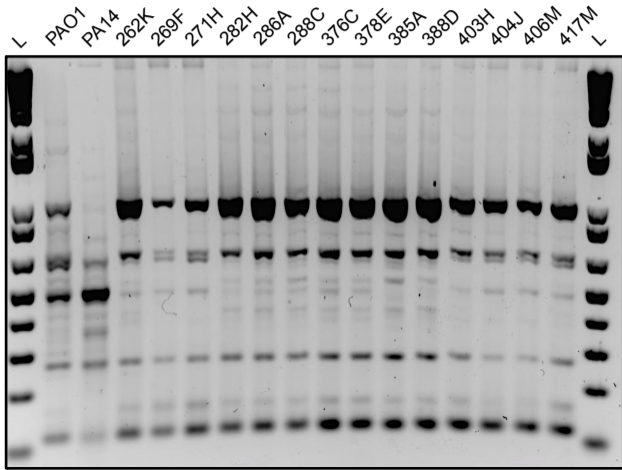

Supplement: Figure S1 [file sph005162141sf1.pdf]
