# Supplementary material for: Environmentally Endemic Pseudomonas aeruginosa Strains with Mutations in lasR Are Associated with Increased Disease Severity in Corneal Ulcers
Source: mSphere. 2016 Sep 7;1(5):e00140-16. doi: 10.1128/mSphere.00140-16 (PMC5014915; doi:10.1128/mSphere.00140-16)
Supplement: Table S1 [file sph005162141st2.docx]

**Table S1. Strains, plasmids and primers used in this study.**

| **Reference No.** | **Strain Name** | **Description** | **Source or Reference** |
| --- | --- | --- | --- |
| ***P. aeruginosa*** |  |  |  |
| DH122 | PA14 WT | Wild-type | (1) |
| DH164 | PA14 ∆*lasR* | In-frame deletion of *lasR* | (2) |
| DH2617 | PA14 LasR P117L | Gene replacement of *lasR* with *lasR* C350T | This study |
| DH2618 | PA14 LasR I215S | Gene replacement of *lasR* with *lasR* T644G | This study |
| DH2619 | WT Revert | Wild-type *lasR* following sucrose-counter selection of the *lasR* T644G merodiploid | This study |
| DH161 | qsc102 | PAO1 *lasI*::Tc *rhlI*::Tn501. *lacZ* integrated into PA1897. Induced by 3OC12HSL | (3) |
| DH2590 | 262K | SCUT clinical isolate | (4) |
| DH2591 | 269F | SCUT clinical isolate | (4) |
| DH2592 | 271H | SCUT clinical isolate | (4) |
| DH2593 | 282H | SCUT clinical isolate | (4) |
| DH2594 | 283J | SCUT clinical isolate | (4) |
| DH2595 | 286A | SCUT clinical isolate | (4) |
| DH2596 | 288C | SCUT clinical isolate | (4) |
| DH2597 | 295K | SCUT clinical isolate | (4) |
| DH2598 | 360J | SCUT clinical isolate | (4) |
| DH2599 | 362M | SCUT clinical isolate | (4) |
| DH2600 | 364B | SCUT clinical isolate | (4) |
| DH2601 | 367E | SCUT clinical isolate | (4) |
| DH2602 | 369G | SCUT clinical isolate | (4) |
| DH2603 | 376C | SCUT clinical isolate | (4) |
| DH2604 | 378E | SCUT clinical isolate | (4) |
| DH2605 | 385A | SCUT clinical isolate | (4) |
| DH2606 | 388D | SCUT clinical isolate | (4) |
| DH2607 | 403H | SCUT clinical isolate | (4) |
| DH2608 | 404J | SCUT clinical isolate | (4) |
| DH2609 | 406M | SCUT clinical isolate | (4) |
| DH2610 | 417M | SCUT clinical isolate | (4) |
| DH2611 | 419B | SCUT clinical isolate | (4) |
| DH2612 | 265B | SCUT clinical isolate | (4) |
| DH2613 | 399D | SCUT clinical isolate | (4) |
| DH2614 | 550A | SCUT clinical isolate | (4) |
| DH2615 | 654F | SCUT clinical isolate | (4) |
| DH2616 | 904C | SCUT clinical isolate | (4) |
| DH2653 | 352A | SCUT clinical isolate | (4) |
| DH2654 | 432D | SCUT clinical isolate | (4) |
| DH2655 | 123C | SCUT clinical isolate | (4) |
| DH2656 | 321C | SCUT clinical isolate | (4) |
| DH2657 | 901M | SCUT clinical isolate | (4) |
| ***E. coli*** |  |  |  |
| DH2419 | SM10 | λ*pir* |  |
| **Plasmids** |  |  |  |
| DH2620 | DH5α | *E. coli*. Shuttle vector pMQ30 for yeast cloning and Gram-negative allelic replacement; Gm^R^ | (5) |
| DH2621 | *E. coli*. pMQ30-derived vector. For gene replacement of *lasR* with LasR P117L variant; Gm^R^ | | This study |
| DH2622 | *E. coli*. pMQ30-derived vector. For gene replacement of *lasR* with LasR I215S variant; Gm^R^ | | This study |
| **Primers** | **Primer sequence (5’-3’)^a^** | |  |
| *lasR_*seq_For | GACGGGTATCGTACTAGGTG | |  |
| *lasR_*seq_Rev | CGAGAATGGCGAGAACCTGC | |  |
| ERIC1R | CACTTAGGGGTCCTCGAATGTA | |  |
| ERIC2 | AAGTAAGTGACTGGGGTGAGCG | |  |
| *lasR_*KON_pMQ30_1 | cagaccgcttctgcgttctgatttaatctgtatcaggctgaCCTGCCGATGACGCCGGCG | |  |
| *lasR_*KON_pMQ30_2 | CTCAAGAAAACCGTCAACCAAGGCCATAGCGCTACGTTCTTCTTAAACTATTAACCAATC | |  |
| *lasR_*KON_pMQ30_3 | ATTGGTTAATAGTTTAAGAAGAACGTAGCGCTATGGCCTTGGTTGACGGTTTTCTTGAGC | |  |
| *lasR_*KON_pMQ30_4 | TCGCCAGCTCGCCGACCTGAGAGGCAAGATCAGAGAGTAATAAGACCCAAATTAACGGCC | |  |
| *lasR_*KON_pMQ30_5 | CCATTATGGCCGTTAATTTGGGTCTTATTACTCTCTGATCTTGCCTCTCAGGTCGGCGAG | |  |
| *lasR_*KON_pMQ30_6 | tgagcggataacaatttcacacaggaaacagctatgCGTAAAGCGCGATCTGGGTCTTGG | |  |

^a^ In primer sequences, upper case letters indicate *Pseudomonas-*specific genomic sequence, and lower case letters indicate sequence identity to the cloning vector.

1. **Rahme LG, Stevens EJ, Wolfort SF, Shao J, Tompkins RG, Ausubel FM.** 1995. Common virulence factors for bacterial pathogenicity in plants and animals. Science **268:**1899-1902.

2. **Hogan DA, Vik A, Kolter R.** 2004. A *Pseudomonas aeruginosa* quorum-sensing molecule influences *Candida albicans* morphology. Mol Microbiol **54:**1212-1223.

3. **Whiteley M, Lee KM, Greenberg EP.** 1999. Identification of genes controlled by quorum sensing in *Pseudomonas aeruginosa*. Proc Natl Acad Sci U S A **96:**6.

4. **Srinivasan M, Mascarenhas J, Rajaraman R, Ravindran M, Lalitha P, Glidden DV, Ray KJ, Hong KC, Oldenburg CE, Lee SM, Zegans ME, McLeod SD, Lietman TM, Acharya NR, Grp SCUT.** 2012. The Steroids for Corneal Ulcers Trial Study design and baseline characteristics. Archives of Ophthalmology **130:**151-157.

5. **Shanks RM, Caiazza NC, Hinsa SM, Toutain CM, O'Toole GA.** 2006. *Saccharomyces cerevisiae*-based molecular tool kit for manipulation of genes from Gram-negative bacteria. Appl Environ Microbiol **72:**5027-5036.
